# Establishment of an* In Vitro* Embryo-Endometrium Model Using Alginate-Embedded Mouse Embryos and Human Embryoid Body

**DOI:** 10.1007/s13770-024-00682-w

**Published:** 2024-11-29

**Authors:** Yoon Young Kim, Yong Jin Kim, Jung Woo Kim, Jiyeon Kim, Sung Woo Kim, Seung-Yup Ku

**Affiliations:** 1 Department of Obstetrics and Gynecology, Seoul National University Hospital, Seoul, Korea; 2 Institute of Reproductive Medicine and Population, Medical Research Center, Seoul National University, Seoul, Korea; 3 Department of Obstetrics and Gynecology, Korea University College of Medicine, Seoul, Korea; 4 Department of Obstetrics and Gynecology, Seoul National University College of Medicine, Daehak-Ro 101, Jongno-Gu, Seoul, 03080 Korea

**Keywords:** Embryo, Embryoid body, Endometrium, Cross-talk, Scaffold

## Abstract

**Background:**

Embryo-endometrium cross-talk is one of the critical processes for implantation, and unsuccessful cross-talk leads to infertility. We established an endometrium-embryo (or embryoid bodies, hEBs)* in vitro* model in 2D and 3D conditions and assessed its potential through the fusion of embryos and the expression of specific markers.

**Methods:**

C57BL/6 mouse embryos and human embryoid body (hEB) derived from embryonic stem cells were prepared as embryo models. Mouse endometrium (EM) and human endometrium cell line, HEC-1-A, were prepared, and 2D or 3D EMs were generated. The viability of the 3D endometrium was analyzed, and the optimal ratio of the gelation was revealed. The invasion of the embryos or hEBs was examined by immunostaining and 3D image rendering.

**Results:**

The embryos and the alternative hEBs were effectively fused into 2D or 3D* vitro* EM models in both mouse and human models. The fused embryos and hEBs exhibited migration and further development. Notably, the established* in vitro* model expressed Oct4 and E-Cadherin, markers for early embryonic development; human CG Receptor and Progesterone Receptor, critical for implantation and pregnancy maintenance; and TSH Receptor, Epiregulin, and Prolactin, indicators of endometrial receptivity and embryo implantation.

**Conclusion:**

This study marks a significant advancement in the field, as we have successfully established a novel* in vitro* model for studying embryo-endometrium cross-talk. This model, a crucial tool for understanding fertility and the causes of miscarriage due to failed implantation, provides a unique platform for investigating the complex processes of successful implantation and pregnancy, underscoring its potential impact on reproductive health.

**Supplementary Information:**

The online version contains supplementary material available at 10.1007/s13770-024-00682-w.

## Introduction

Implantation is the first stage of pregnancy and refers to a series of processes in which the embryo developed after fertilization reaches the uterine cavity through the fallopian tube and undergoes apposition, adhesion, and invasion into the endometrium in the blastocyst state [1]. The human endometrium proliferates by estrogen produced in the ovarian follicles, then becomes receptive to the embryo through secretory differentiation and decidualization by progesterone (P4) secreted by the corpus luteum after ovulation [2, 3]. The human endometrium is structurally and functionally most capable of accommodating an embryo during the mid-luteal phase, referred to as the window of implantation (WOI) [4]. Synchronization of female reproductive hormones periodically secreted from the ovaries opens the WOI and enables successful embryo implantation. However, even during the window of implantation, not all embryos are successful in implantation. Successful implantation requires both the blastocyst to acquire the ability to implant and the endometrium to be receptive. For this, molecular cross-talk between the embryo and the endometrium is essential [1]. Listening to the molecular cross-talk between the human embryo and the endometrium is the key to uncovering the mechanisms of implantation and solving infertility.

Due to ethical and technical difficulties in collecting and analyzing endometrial tissue from women in the first trimester of pregnancy, we do not yet fully understand the molecular mechanisms that occur at the moment of implantation in the microenvironment between the human embryo and endometrium [5]. Alternatively, studies have collected endometrial tissue from the cycle before embryo transfer in infertile patients, analyzed gene expression patterns to confirm WOI, and assessed endometrial receptivity to determine a personalized embryo transfer [6, 7]. However, there are limitations to its clinical application, including reproducibissues due to the use of endometrial samples from previous cycles rather than embryo transfer cycles. While animal models have provided critical biological clues to the physiology of implantation in the endometrium, mouse models do not fully reflect the human implantation process [8]. Human embryos invaginate endometrial epithelial cells with the inner cell mass (ICM) facing the apical surface of the luminal epithelium. In contrast, mouse embryos invaginate endometrial epithelial cells through apoptosis or entosis, with the ICM facing the luminal side of the uterus [1, 9]. Unlike humans, the mouse endometrium does not undergo decidualization of stromal cells, nor does it undergo the periodic shedding of the endometrium, menstruation, and regeneration [10]. Therefore, it is essential to establish an* in vitro* model that recapitulates the interface between the human embryo and the endometrium to accurately and in-depth understand the implantation process of human embryos.

Early* in vitro* models for studying the mechanisms of implantation of human embryos utilized two-dimensional (2D) culture methods in which endometrial cells were cultured in plastic dishes coated with a collagen monolayer [11]. In the case of the uterus, three-dimensional (3D) culture models are essential for studying implantation mechanisms, as stromal cells and their surrounding extracellular matrix (ECM), as well as epithelial cells of the endometrium, play a crucial role in embryo invasion during implantation [12]. The ECM provides mechanical support for epithelial cells and induces the expression of receptivity-related molecules on the apical membrane of epithelial cells in response to changes in female reproductive hormones during the menstrual cycle [13]. In addition, it induces changes in the polarity of epithelial cells. It prepares for embryo implantation by weakening lateral cell–cell and cell–matrix adhesion through changes in junctions and adhesion molecules in the basolateral membrane of epithelial cells [14]. However, 2D cultures of endometrial cells lack an ECM, which makes it difficult to mimic the physiological functions that occur* in vivo* entirely, and they are less responsive to external environments, such as changes in female reproductive hormones. To overcome the limitations of 2D cell culture, 3D cell culture models have been introduced. Still, previous studies have used immortalized cell lines or cancer cells, so it is difficult to assume that they simulate healthy endometrium with normal function [15–17]. Furthermore, there is a limitation that the model was established, and the competence of the established model, i.e., implantation function, was not validated by co-culturing the embryos [18–21].

In this study, we established an* in vitro* 3D model that simulated the interface between the embryo and endometrium during implantation of a human blastocyst and confirmed that the established model could accommodate mouse embryos and human embryoid bodies (EBs). This model will provide novel basic knowledge in understanding the implantation mechanism of human embryos and contribute to the development of infertility treatments by expanding the understanding of the pathophysiology of diseases such as repeated implantation failure and uterine factor infertility.

## Materials and methods

### Ethics for animal and human embryonic stem cell

#### Animal experiments

The Seoul National University Hospital’s institutional animal care and use committee (IACUC) reviewed and approved all animal experiments [IACUC approval No. 20-0033-S1A3(1)].

#### Human embryonic stem cell experiments

The institutional review board (IRB) of the Institute of Reproductive Medicine and Population, Seoul National University Medical Research Center [IRB approval No.219932-202205-05-01-01] reviewed and approved human embryonic stem cell (hESC)- related experiments and the usage of the hESC line, SNUhES34.

### Mouse and human endometrial cell culture

7-week-old C57BL/6 female mice (Koatech, Gyeonggi-do, Korea) were sacrificed by cervical dislocation. The uterus was isolated, and lipid pads were removed. Isolated uteri were mechanically dissected and dissociated using collagenase type I (5 mg/ml) for 90 min. Dissociated cells were filtered through a 70-um cell strainer (SPL Life Sciences, Gyeonggi-do, Korea) and centrifugated at 2000 rpm for 10 min. Collected cell pellets were resuspended in culture media and replated. Culture media constituted with DMEM/F12, supplemented with 10% fetal bovine serum (FBS), 1% Insulin-Transferrin-Selenium-X (ITS), and 100 units/ml of penicillin-100 ug/ml of streptomycin. The medium was changed every three days. When the cells reached approximately 90% confluence, they were passaged using 0.25% trypsin–EDTA.

The HEC-1-A cell line for human endometrial cells was purchased from ATCC (HTB-112, Manassas, VA, USA). The cells were grown in the same media as mouse endometrial cells. Invitrogen purchased all the reagents for mouse and human endometrial cell cultures.

### Production of mouse embryos

#### Superovulation

6-week-old C57BL/6 female mice were injected into the peritoneal cavity with 10 IU/0.1 ml of pregnant mare serum gonadotropin (PMSG, Prospec, East Brunswick, NJ, USA). At 48 h later, 10 IU/0.1 ml of human chorionic gonadotropin (hCG, Sigma-Aldrich, Saint Louis, MO, USA) was intraperitoneally administered. The mice were sacrificed by cervical dislocation at 20 h after hCG injection.

#### Preparation of sperm

A dense sperm mass was obtained from the epididymis of C57BL/6 mice 8–10 weeks old. The sperm in an HTF media (Cosmo Bio, Carlsbad, CA, USA) drop of 100 ul covered with mineral oil was incubated at 37 °C in 5% CO_2_ in humidified air for 1 h to obtain fertilization ability.

#### Oocyte collection

Oocytes were obtained from oviducts at 20 h after hCG administration. The oviducts were isolated and placed in a 100 ul of TYH medium (Cosmo Bio) droplet in a dish. The cumulus-oocyte complexes were isolated from the swollen ampulla and transferred to the TYH medium. Then, oocytes were incubated for about 1 h at 37 °C in 5% CO2 in a humidified air atmosphere.

#### *In vitro* fertilization (IVF)

A pre-incubated, capacitated sperm was gently added to the freshly ovulated oocytes for a final motile sperm concentration of 1 × 10^6^/ml. The combined sperm oocytes were incubated for 5 h. Then, the oocyte was washed through several medium changes and incubated in 40 ul of mWM media (Cosmo Bio) drop covered with mineral oil. Development of 2 cell embryos was counted 24 h after the completion of* in vitro* fertilization. The image of embryos was recorded using the *i*-solution program (IMT i-Solution, Daejeon, Korea).

### Human embryonic stem cells and embryoid bodies

#### Human embryonic stem cell (hESC) culture

The human embryonic stem cell line, SNUhES34 (46, XX), was provided by the Institute of Reproductive Medicine and Population, Seoul National University. The hESC line was maintained in feeder-free condition. Five ug/ml of vitronectin (VTN) was pre-coated at room temperature (RT) for 1 h. Full-grown colonies were incubated with 0.5 mM of EDTA for 5 min, and the reaction stopped. The colonies were detached and split with gentle pipetting into small clumps. The clumps were distributed evenly to the VTN pre-coated dish. Essential 8 medium was used for the culture, and the media was exchanged daily.

#### Human embryoid body (hEB) formation

Undifferentiated hESCs were treated with collagenase Type IV (2 mg/ml) and incubated for 30 min at 37 °C. The dissociated colonies were dissociated into small clumps and incubated overnight in a suspension culture. hEBs were incubated for 48 h and used as a human embryo alternative model. All the reagents for hESCs and hEBs were purchased from Invitrogen.

### Endometrium-3D gel mixture preparation

Mouse endometrium cells at a density of 3 × 10^7^ cells/well were mixed with pre-warmed Bio-Gel (Medipab, Seoul, South Korea) at 37 °C in ratio 1:1, 2:1 and 3:1 (Cells with media: Bio-Gel). Four hundred ul/well of the mixture were seeded in 4 well plates (SPL) and gelated at 4 °C overnight, and Casting gel (Medipab) was added for secondary gelation at 37 °C for 3 h. After washing with media, the firmed gel mixture was covered with 200ul/well of culture media. The cell mixture with gel was treated with a firming buffer every two days to harden it extra, and the media was changed. It was cultured for 1, 3, 5, 7, and 9 days.

### Cell survival assay

For viability measurement, 3D-embedded, cultured cells were treated with a dissolving solution for 30 min at RT. The CCK-8 component was added to the cells and incubated for 2 h at 37 °C, 5% CO_2._ The absorbance of treated cells was measured at 450 nm using a microplate reader device (Molecular Devices, San Jose, CA, USA).

### Fluorescence-activated cell sorting (FACS)

Cells were dissociated into single cells by treating Accutase (Invitrogen) for 5 min at 37 °C. Dissociated cells were incubated with blocking solution (PBS with 3% BSA and 0.3% Triton X-100, All from Sigma-Aldrich) for 30 min at RT and washed with PBS. The samples were incubated with conjugated antibodies for 1 h at 37 °C. Then, the samples were washed with PBST three times and analyzed by FACS Calibur™ (BD Biosciences, Franklin Lakes, NJ, USA). IgG antibody was used as the control for the analysis. Antibodies used for the study are listed in Supplementary Table 1.

### Immunostaining

The samples were fixed with 4% paraformaldehyde for 20 min at RT. The samples were washed with PBS and blocked for over 12 h using blocking soln. Primary antibodies were treated for 1 h at 37 with a ratio of 1:100. After three times of washing with PBST, secondary antibodies were treated for 1 h at 37 °C with a ratio of 1:200. And then, the samples were cleaned using PBST and mounted with Prolong Gold containing DAPI (Invitrogen). Antibodies used for the analysis are listed in Supplementary Table 2 and purchased from Abcam (Waltham, MA, USA) and Santa Cruz Biotechnology (Dallas, Texas, USA).

### Statistics

Statistical analysis was performed using Prizm GraphPad version 9.0 (GraphPad Software, San Diego, CA, USA). Data are expressed as the mean ± standard errors and were analyzed by one-way ANOVA. *P* value less than 0.05 (*P* < 0.05) is considered statistically significant.

## Results

### Characterization of endometrial cells (EM) according to passage

**Fig. 1:**
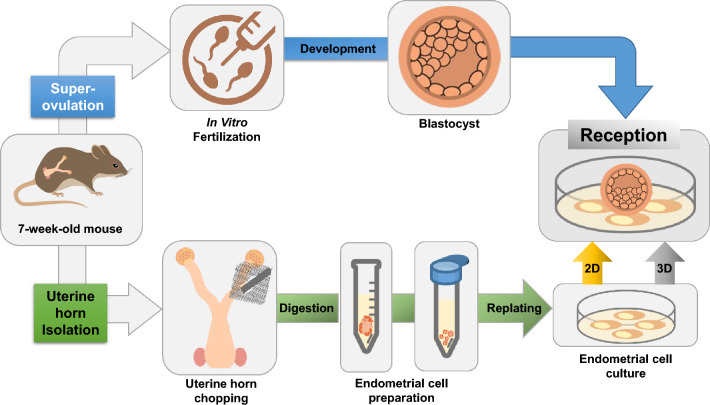
Experimental scheme of mouse model

The experimental scheme of mouse endometrium embryos is presented in Fig. 1. Isolated endometrial cells were maintained* in vitro* for less than four passages without the addition of any hormones.* In vitro* cultured EM were used for further analyses and production of 3D endometrium.

**Fig. 2:**
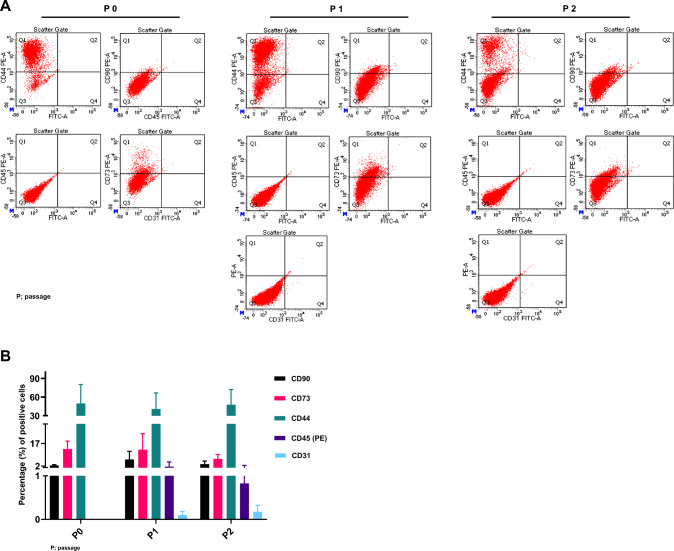
Maintenance of characteristics during prolonged* in vitro* culture. **A** Expressions of CD31, CD44, CD45, CD73, and CD90 and their alterations after* in vitro* cultures were analyzed. **B** Proportion of specific markers after prolonged* in vitro* culture in mouse endometrial cells. P: passage

As well-known alterations during* in vitro* culture, the characteristics of endometrial cells were analyzed as they passaged. The expression of CD 31, CD44, CD45, CD73, and CD91 were maintained similar from passage number (PN) 0–2 (Fig. 2A). Expression of CD 31, − 45, and − 90 were merely detective and CD44 and CD73 were positively expressed. The positive percentages of CD44 and CD73 were similar in each PN (Fig. 2B). These results indicated that the isolated endometrial cells maintained their characteristics during* in vitro* expanded culture.

### Fusion of embryo on* in vitro* cultured endometrium

**Fig. 3:**
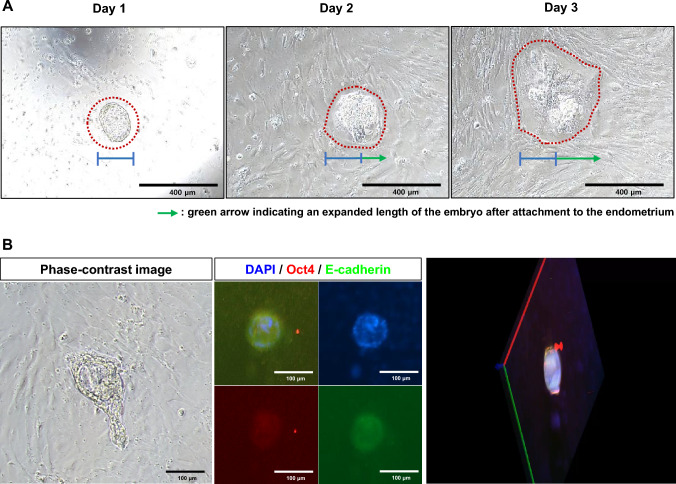
Fusion of embryo on* in vitro* cultured endometrium. **A** The embryo was attached to the endometrium at day 1. The fused embryo changed its morphology when observed on day two and further developed on day 3. The red line indicates the developing embryo. The blue line indicates the diameter of the embryo on day 1, and the green arrow indicates an expanded length of the embryo after attachment to the endometrium. **B** Expressions of embryo-specific markers in attached embryos and their rendering moving image. The attached embryo maintained its characteristics as an embryo and expressed specific markers, Oct4 (red) and E-cadherin. The attachment and invasion into the endometrium were analyzed by stacking rendering images

To assess the fusion of embryos on endometrium, embryos were produced by* in vitro* fertilization (IVF, Supple Fig. 1A). For the mass production of the embryo, TYH and HTF medium were used, and the efficiency was not significantly different (Supple Figs. 1B and 2C). Based on this result, we used the TYH medium in further experiments. IVF-created embryos were developed into early blastocysts and transferred to grown endometrial cells. The fusion of embryos was monitored every 6 h. The attached fused embryos’ morphology changed as they developed. The diameter of an embryo was expanded twice from day 1 to 3 (Fig. 3A). Fused embryos expressed early embryo stage-specific genes such as Oct4, the specific markers of pluripotency in the inner cell mass (ICM) of the blastocyst. The expression of Oct4 was restricted in the ICM region. In addition, embryo-specific ECM marker E-cadherin was also expressed in the embryo, and the region of expression was out a layer of the embryo (Fig. 3B). These results indicated that the embryos were fused with endometrium with a similar phenomenon* in vivo* and reception of embryo in EM might be mimicked* in vitro*.

### Expressions of embryo-receptivity-related markers

**Fig. 4:**
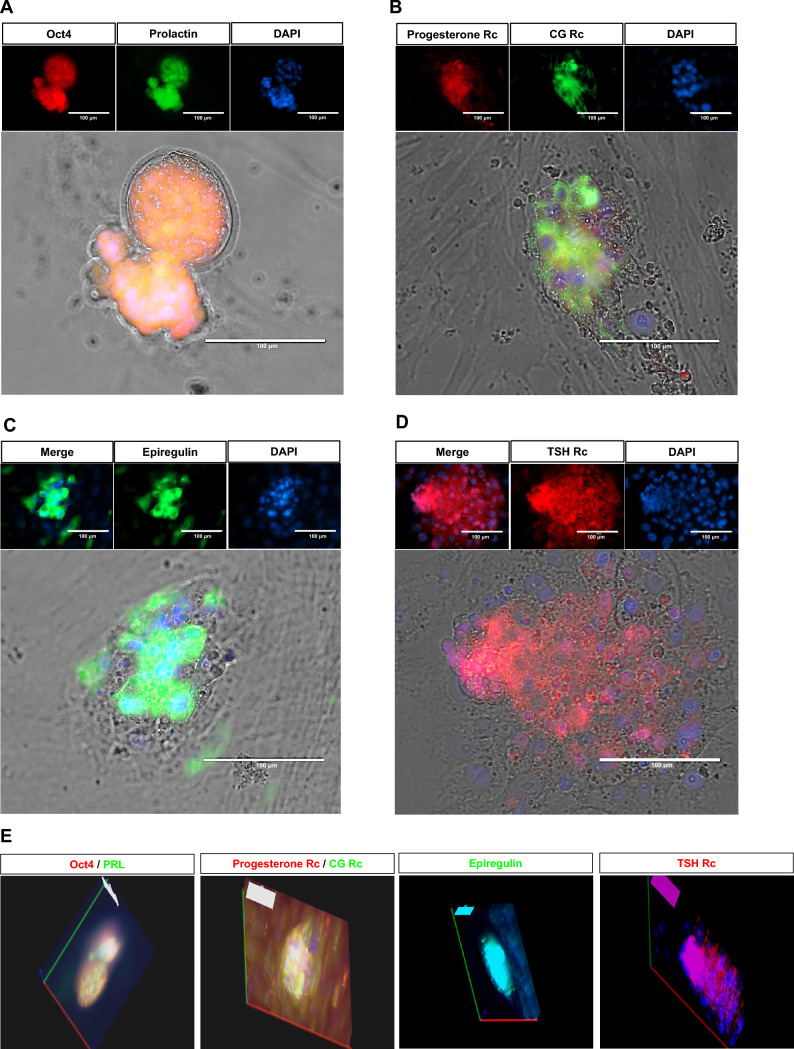
Expressions of embryo-receptivity markers in a fused mouse 2D model. **A** Expressions of Oct4 and Prolactin in endometrium attached, hatched embryo. **B** Expressions of Progesterone and Chorionic Gonadotropin (CG) receptors in embryo-fused mouse endometrium. **C** Expression of Epiregulin in fused embryo. **D** Thyroid Stimulating Hormone Receptor (TSH) receptor was also expressed in embryo-fused endometrium. **E** Collection of stacked rendering images of specific markers in embryo-fused, mouse 2D endometrium model. Rc: receptor

In the embryo-endometrium fusion model, embryo and receptivity-specific markers were analyzed. In the hatched blastocyst, Oct4, the specific marker of ICM of the embryo, and prolactin (PRL), the marker of implantation, were highly expressed and demonstrated the complete fusion (Fig. 4A). To confirm the receptivity, the expressions of progesterone receptor and chorionic gonadotropin (CG) receptors were evaluated. Embryo-fused endometrium expressed the receptors of progesterone and CG (Fig. 4B). Additionally, the expressions of epiregulin and thyroid-stimulating hormone (TSH) receptor, which is known as a regulator of the implantation process (Fig. 4C and D). The 3D rendering images revealed the fusion of embryos into the endometrium (Fig. 4E) and expressions of the implantation-specific markers.

### Production of 3D endometrium alginate gel

**Fig. 5:**
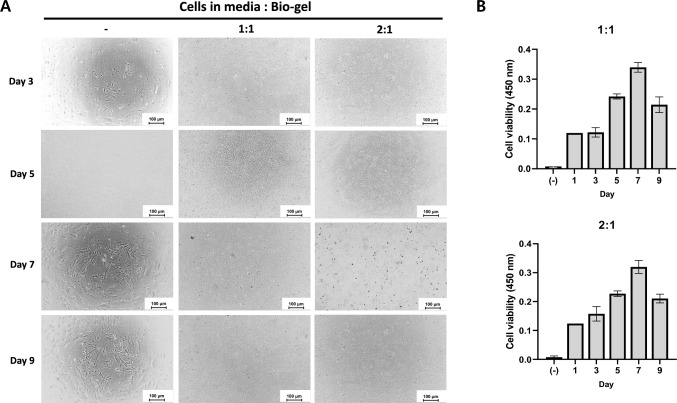
Constitution of 3D endometrium using alginate. 3D endometrium gels were produced under the various cell-to-3D matrix ratios. **A** Morphologies of the different ratio 3D endometrium gels after formation. **B** Cell viabilities of 1:1 and 2:1 gel

For the construction of 3D endometrium, the optimal ratio of the cell to alginate gel was determined. The mixture ratio of the cells affected the gel maintenance. The 1:1 and 2:1 mixture gel remained formed for up to 9 days (Fig. 5A); however, the 3:1 mixture gel was not maintained for as long as 1:1 and 2:1 (Supple Fig. 2A and B). The mixture demonstrated the growth of embedded endometrial cells by measuring cell viability. The viability of the 3D EM-gel was increased up to 7 days in both ratios, and the viability decreased after 7 days (Fig. 5B). These results indicated that the alginate-embedded 3D endometrial gel remained proliferative and possibly applied as 3D* in vitro* endometrium model.

### Invasion of embryo on 3D endometrium

**Fig. 6:**
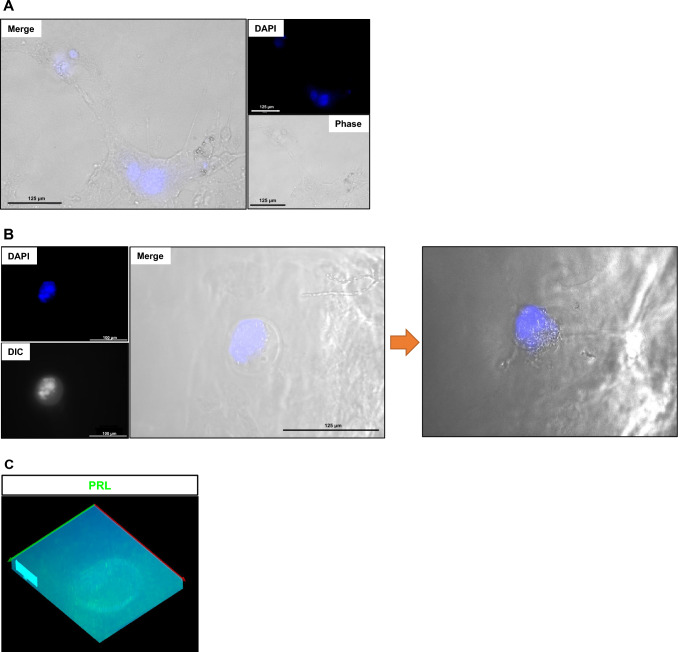
Invasion of the embryo into 3D endometrium. Developed embryos were attached to 3D endometrium gel, and their invasion was analyzed. **A** Localization and movement of a fused embryo were confirmed by DAPI staining. **B** Fusion of the ICM in the embryo to 3D endometrium

The invasion of mouse embryos in 3D endometrium was monitored every 24 h and the ICM of the embryo spread out into the 3D endometrium (Fig. 6A). The Inner cell mass of the embryos was transiently localized in a region of 3D endometrium and fused with 3D endometrium (Fig. 6B, left panel). The ICM region of the embryo was hatched out and fused with 3D endometrium (Fig. 6B, right panel). Expression of PRL was confirmed in embryos fused in 3D endometrium gel (Fig. 6C).

### Human embryoid body-endometrium fusion 2D model

**Fig. 7:**
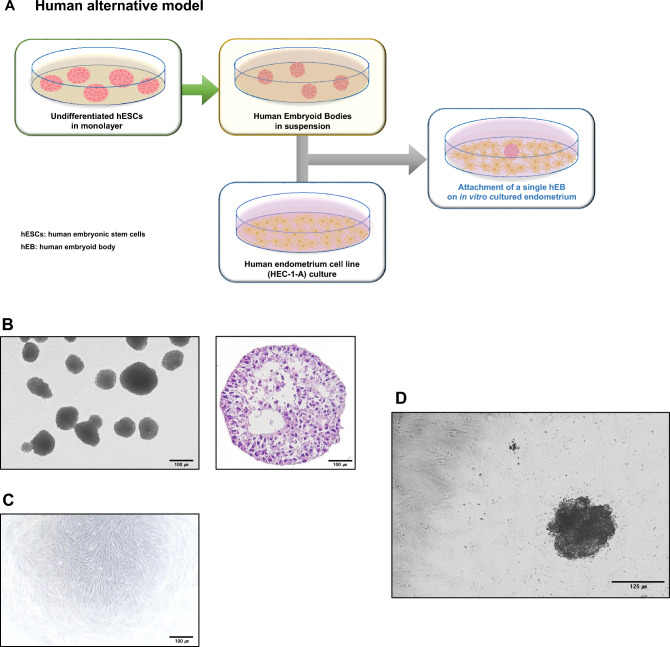
Production of human embryoid body (hEB)-endometrium fused 2D model. **A** Experimental scheme of a human alternative model. **B** Morphologies of hESC-derived hEBs (left panel), intra structure of hEBs (right panel). **C** Morphology of the human endometrial cell line. **D** The hEB attached to human endometrial cells

The experimental scheme of the human endometrium-embryoid body (hEB) model is presented in Fig. 7A. Human embryonic stem cell-derived EB was used as a human embryo alternative model. A single human embryoid body (Fig. 7B) was placed onto a pre-cultured human endometrial cell line (Fig. 7C), and their fusion was observed (Fig. 7D). The structure of hEBs showed a similar structure to the early embryo. Characteristics of undifferentiated human embryonic stem cells were demonstrated by the expression of Oct4 and Tra-1–81 (Supple Fig. 3).

## Discussion

Receptivity of the endometrium to the embryo is the critical process of successful implantation. If this receptivity fails, it results in infertility, including repeated pregnancy loss, which is one of the primary reasons for infertility. The receptivity relies on the cross-talk between endometrium and embryo, and various species models were established to mimic the process [22–28]. Due to the complexity of the process and ethical limitations of using human embryos, establishing an* in vitro* model is rare despite their necessity [29]. In addition, the reception of the embryo consists of sequential steps, including apposition, adhesion, and invasion. Therefore, the 3D model is superior from the* in vitro* receptivity and observation perspective. However, studies of proper scaffolds that are non-toxic and do not interrupt the invasion of the embryo are still limited [30–33].

In this study, we tried to establish an* in vitro* endometrium-receptivity model in 2D and 3D conditions using mouse and human alternatives, the hEB model. We tested three endometrium ages for endometrium preparation: immature (2W), nulliparous, and parous EM. Nulliparous EM of 7–8 weeks was well-grown* in vitro* without hormone treatment. The EMs were passaged in less than four passages, and their characteristics were maintained (Fig. 2A). CD33 and CD44 were positively expressed, and the CD31, CD45, and CD90 were not expressed as previously reported [34].

IVF-generated embryos are attached to the endometrium, and the embryo is fused with the endometrium and further developed (Fig. 3A). The fused embryo changes its morphology, and the diameter is reached about two times. At this stage, we analyzed the expressions of embryo and receptivity markers. The ICM-specific marker, Oct4, and prolactin were also expressed in fused, hatched embryos. Receptivity-specific markers were also highly expressed in embryo-fused endometrium (Fig. 4A–D). Fused region of embryo-EM expressed progesterone receptor, CG receptor, TSH receptor, and Epiregulin. 3D rendering images demonstrated the invasion of the attached embryo and the movement of embryo-derived materials. These results showed that the embryo fused with EM further developed and expressed receptivity markers in the 2D model. This model could be applied as an* in vitro* model of embryo-receptivity model.

As mentioned above, we established 3D EM using alginate. Combining EM and alginate gel was necessary to construct 3D EM. A 2:1 ratio was determined as a proper ratio for cells in media to bio-gel. Embryos attached and fused to 3D EM and migration in the 3D endometrium were confirmed (Fig. 6A and B). The results indicated that the mix ratio of EM to scaffold is critical for constructing 3D EM.

We used a human embryonic stem cell-derived embryoid body (hEB) to construct a human alternative model [35, 36]. Embryoid bodies are three-dimensional aggregates of stem cells that can mimic the early stages of embryo formation, including the development of the three germ layers: ectoderm, mesoderm, and endoderm [37]. This model is valuable because it offers insights into how human embryos develop in the early stages, which can be challenging to study directly due to ethical and practical limitations [38]. hEBs were successfully fused to 2D and 3D EM-derived from humans. The fused hEBs developed further by migration. This alternative model can provide crucial information about normal development and embryo-endometrium cross-talk.

This study investigates the embryo-endometrium cross-talk in* in vitro* mouse and human models. Proper scaffolds for EM were elucidated, and their optimal ratio was revealed. The attached embryos survived and migrated into the endometrium. In addition, the fused embryos expressed receptivity-specific markers. Furthermore, we tried to use the human embryoid body as an alternative human embryo model [39]. However, this study has some limitations due to the limited control over the development degree of hEBs. While hEBs can mimic certain aspects of embryogenesis, they lack the precise developmental cues and spatial organization found in an* in vivo* embryo, leading to spontaneous and sometimes uncontrolled differentiation. This results in a developmental stage that does not perfectly align with that of a natural human embryo. To address these limitations, ongoing research aims to develop better methods to guide and control the differentiation of hEBs, making them more reliable models for studying human development. Furthermore, dual approaches using non-human primates resembling human reproductive physiology may enhance overcoming the limitations [40–44].

In this study, we established mouse and human embryo-endometrium cross-talk *in vitro* models that simulate the interaction between the embryo (or embryoid bodies, hEBs) and the endometrium, a critical aspect of early pregnancy. By establishing both 2D and 3D conditions for these models, we have likely aimed to better replicate the complex environment of the uterus during early embryo implantation. These models could provide significant insights into potential treatments for infertility and miscarriage.* In vitro* models that recreate early human embryonic development allow researchers to closely observe processes like fertilization, blastocyst formation, and implantation. Understanding the key stages can identify potential interventions and improve assisted reproductive technologies like IVF. Advanced* in vitro* models that simulate the endometrial lining (such as 3D endometrial models) allow researchers to identify specific factors that facilitate or inhibit molecular interactions of implantation, it may be possible to develop drugs or treatments that enhance implantation, reducing infertility and the chances of miscarriage.

Overall, this work contributes to developing more sophisticated* in vitro* models for studying human reproductive biology, which could have implications for understanding and treating fertility issues.

## Supplementary Information

Below is the link to the electronic supplementary material.

Supplementary file1 (PPTX 25144 KB)

Supplementary file2 (DOCX 15 KB)

## Data Availability

The data supporting this study’s findings are available from the corresponding author upon reasonable request.
