# Supplementary material for: Establishment of an In Vitro Embryo-Endometrium Model Using Alginate-Embedded Mouse Embryos and Human Embryoid Body
Source: Tissue Eng Regen Med. 2024 Nov 29;22(1):77–89. doi: 10.1007/s13770-024-00682-w (PMC11711978; doi:10.1007/s13770-024-00682-w)
Supplement: Supplementary file 1 — Supplementary file1 (PPTX 25144 KB) [file 13770_2024_682_MOESM1_ESM.pptx]

## Slide 1
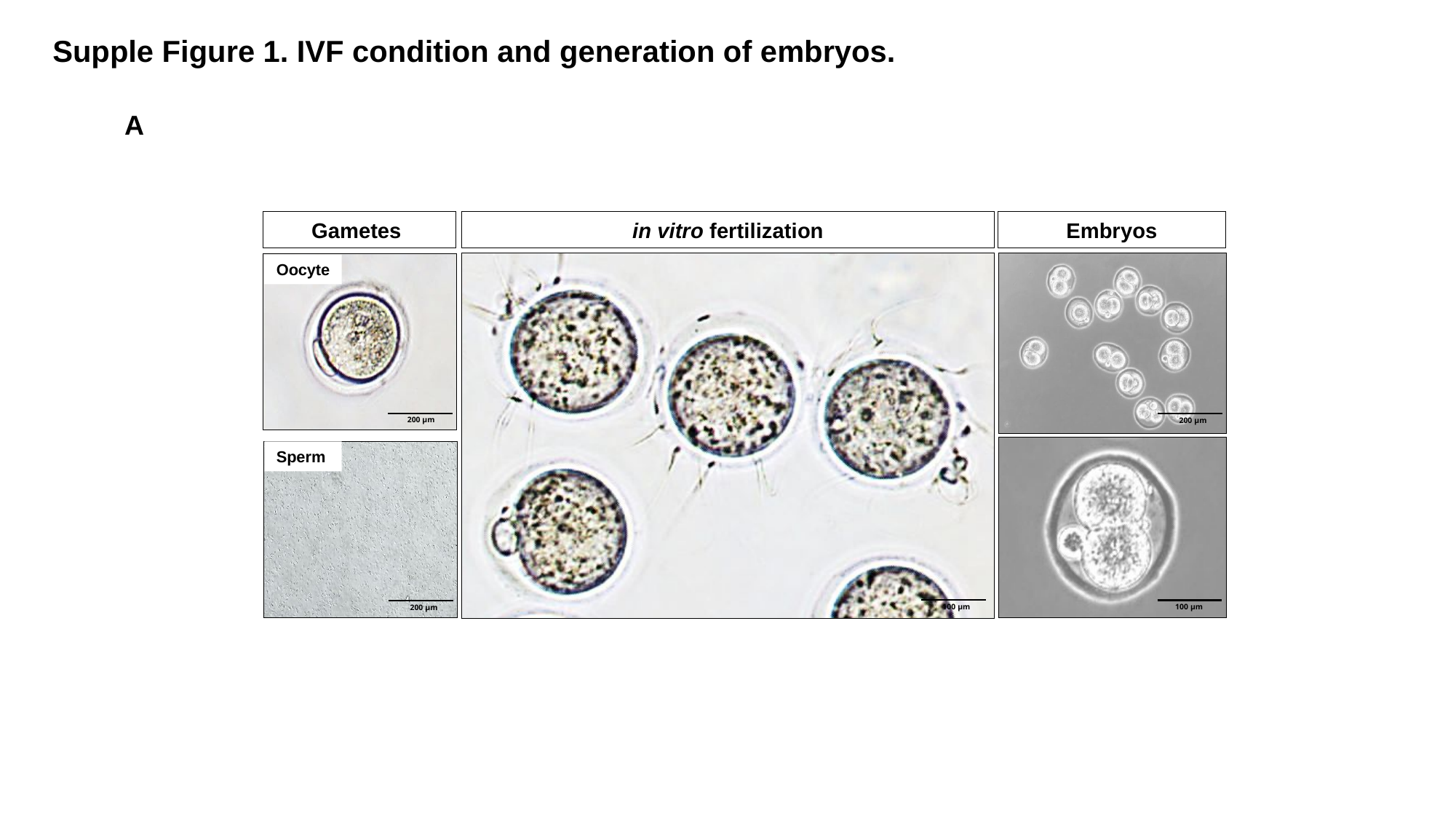

Supple Figure 1. IVF condition and generation of embryos.
A
Embryos
in vitro fertilization
Gametes
Oocyte
200 μm
200 μm
Sperm
100 μm
100 μm
200 μm

## Slide 2
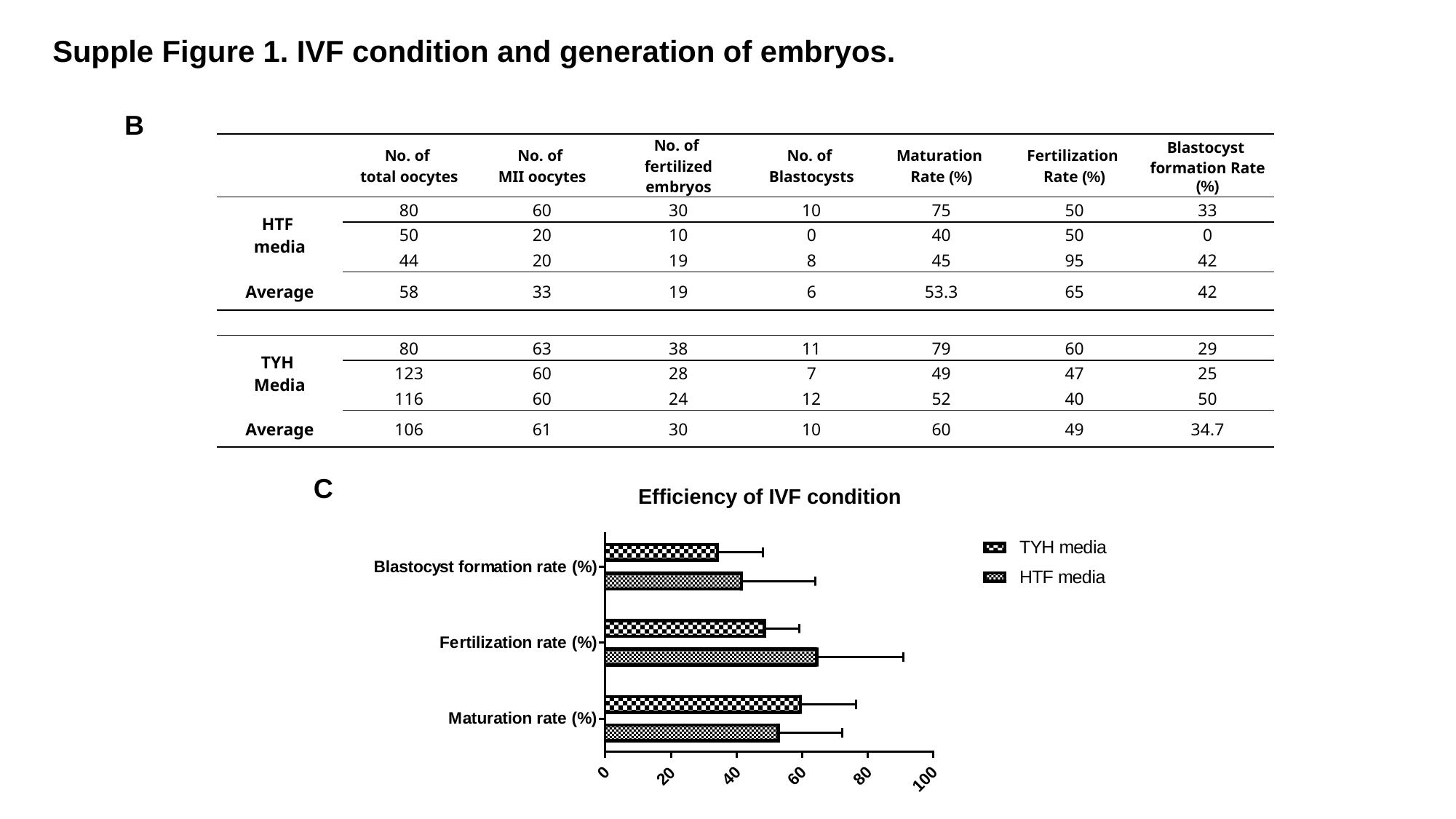

Supple Figure 1. IVF condition and generation of embryos.
B
| | No. of total oocytes | No. of MII oocytes | No. of fertilized embryos | No. of Blastocysts | Maturation Rate (%) | Fertilization Rate (%) | Blastocyst formation Rate (%) |
| --- | --- | --- | --- | --- | --- | --- | --- |
| HTF media | 80 | 60 | 30 | 10 | 75 | 50 | 33 |
| | 50 | 20 | 10 | 0 | 40 | 50 | 0 |
| | 44 | 20 | 19 | 8 | 45 | 95 | 42 |
| Average | 58 | 33 | 19 | 6 | 53.3 | 65 | 42 |
| | | | | | | | |
| TYH Media | 80 | 63 | 38 | 11 | 79 | 60 | 29 |
| | 123 | 60 | 28 | 7 | 49 | 47 | 25 |
| | 116 | 60 | 24 | 12 | 52 | 40 | 50 |
| Average | 106 | 61 | 30 | 10 | 60 | 49 | 34.7 |
C

## Slide 3
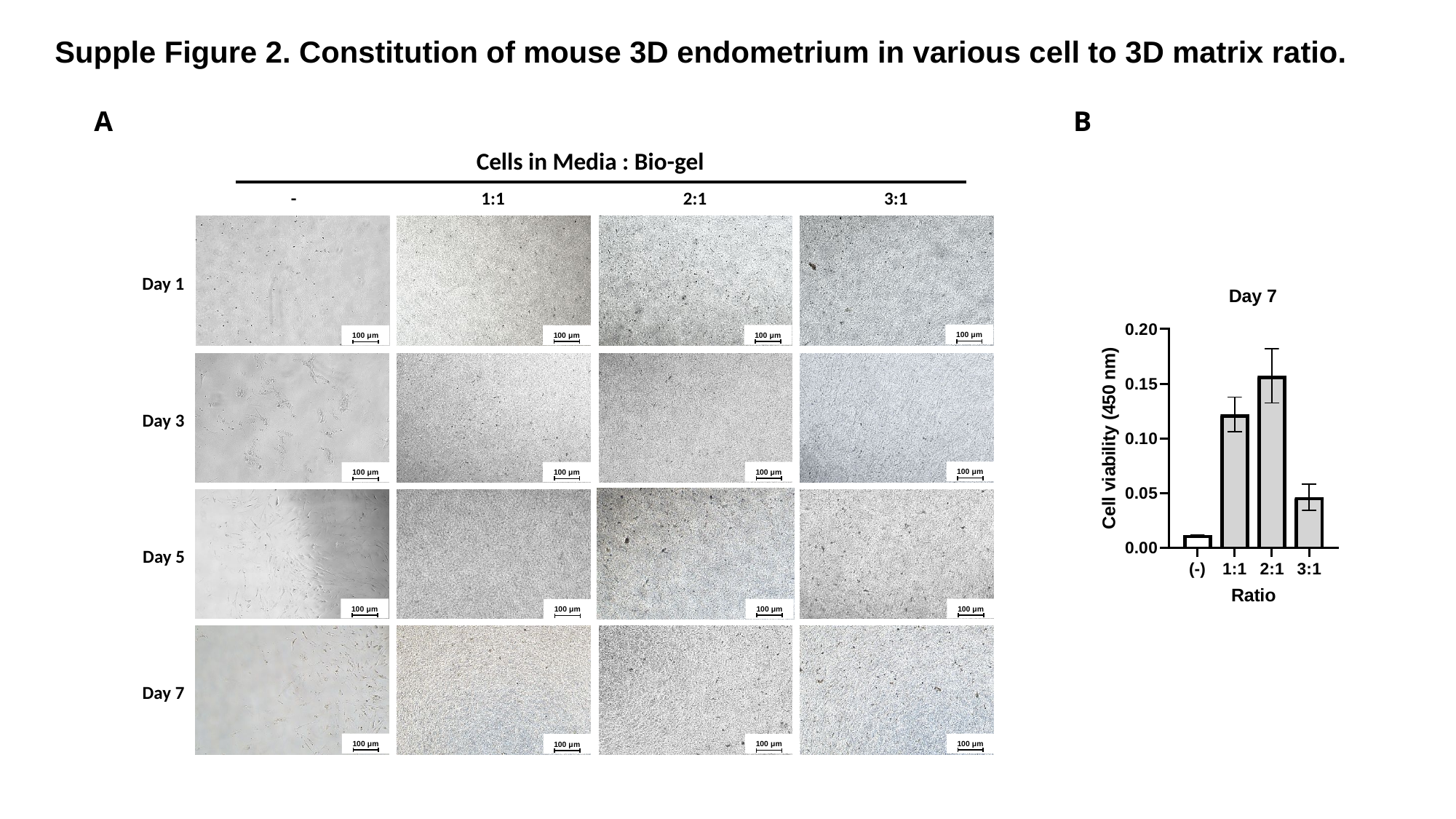

Supple Figure 2. Constitution of mouse 3D endometrium in various cell to 3D matrix ratio.
A
B
Cells in Media : Bio-gel
-
1:1
2:1
3:1
Day 1
100 μm
100 μm
100 μm
100 μm
Day 3
100 μm
100 μm
100 μm
100 μm
Day 5
100 μm
100 μm
100 μm
100 μm
Day 7
100 μm
100 μm
100 μm
100 μm

## Slide 4
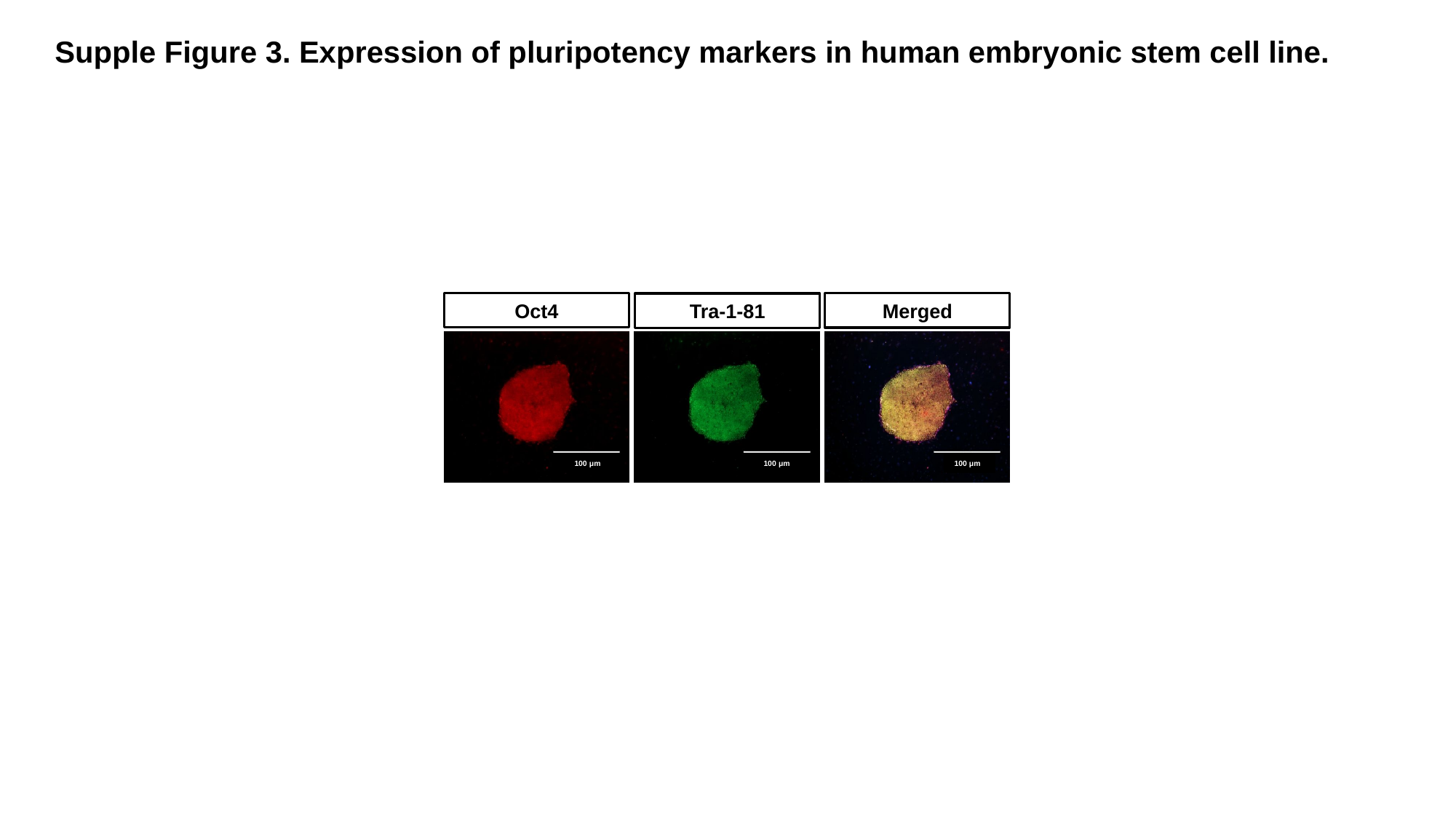

Supple Figure 3. Expression of pluripotency markers in human embryonic stem cell line.
Oct4
Merged
Tra-1-81
100 μm
100 μm
100 μm
