# Supplementary material for: Establishment of an In Vitro Embryo-Endometrium Model Using Alginate-Embedded Mouse Embryos and Human Embryoid Body
Source: Tissue Eng Regen Med. 2024 Nov 29;22(1):77–89. doi: 10.1007/s13770-024-00682-w (PMC11711978; doi:10.1007/s13770-024-00682-w)
Supplement: Supplementary file 2 — Supplementary file2 (DOCX 15 KB) [file 13770_2024_682_MOESM2_ESM.docx]

**Supplementary Tables**

**Supplementary Table 1. List of antibodies used for FACS analysis.**

| **Name** | **Concentration** | **Cat. No.** | **Company** |
| --- | --- | --- | --- |
| CD31 FITC-conjugated | 1:50 | 555445 | BD Pharmingen |
| CD44 PE-conjugated | 1:50 | 550989 | BD Pharmingen |
| CD45 PE-conjugated | 1:50 | 561866 | BD Pharmingen |
| CD73 PE-conjugated | 1:50 | 550257 | BD Pharmingen |
| CD90 PE-conjugated | 1:50 | 555596 | BD Pharmingen |

**Supplementary Table 2. List of primary antibodies used for immunostaining.**

| **Name** | **Concentration** | **Cat. No.** | **Company** |
| --- | --- | --- | --- |
| human CG Receptor | 1:100 | ab204950 | Abcam |
| E-Cadherin | 1:100 | ab76055 |  |
| Oct4 | 1:100 | ab184665 |  |
| Progesterone Receptor | 1:100 | ab2765 |  |
| Tra-1-81 | 1:100 | ab16289 |  |
| TSH Receptor | 1:100 | ab202960 |  |
| Epiregulin | 1:100 | SC-376284 | Santa Cruz Biotechnology |
| Prolactin (PRL) | 1:100 | SC-365659 |  |
